# Legal Notes for Institutional Workers

**Published:** 1919-11-01

**Authors:** 


### LEGAL NOTES FOR INSTITUTIONAL WORKERS.

### A Hard Case.

*Doolan* v. *Hope and Sons*, 145 L. T. J. 26. was an entirely novel case under the Workmen's Compensation Act, and one that is medically of much interest. The workman had his eye injured by a chip of brick. It was examined by a fellow-workman, and he did no more work that day, but went home, where it was examined by his mother and brother. No substance was found in the eye or in the eyelid. In the afternoon his eye became a little better, but in the evening it seemed worse, and he consulted a chemist, who supplied a lotion which was duly applied. Two days later the workman's eye was discharging, and he went to an eye hospital. He was examined by a doctor there, who found the eyelid very much swollen and there was a copious discharge from the eye. The doctor caused some of the discharge to be examined by a pathologist, and it appeared that it was due to gonorrhœa infection, the *coccus* of gonorrhœa being readily identified. All the medical witnesses—the pathologist, a surgeon to the eye hospital, and an eye specialist—agreed that the workman was suffering from gonorrhœa infection of the left eye. The infection spread to the right eye, but ultimately that eye was cured by appropriate treatment. The workman, however, lost the sight of his left eye. He was examined both for syphilis and gonorrhœa, but no trace of the former disease was discovered, nor was there anything to show that the workman had suffered from the latter except when on this particular occasion his left eye became infected. In the proceedings by the workman for compensation the County Court Judge found that the sources from which the workman's eye might have become infected were by the touch of the persons by whom it was examined before he went to the hospital, or by contact with some infected article, such as a handkerchief, a sponge, or a towel, and such article might have been used at the employer's works or the workman's home. He held, and on appeal his judgment was affirmed, that the workman was not entitled to compensation, as the destruction of the eye was not due to the original accident, nor even by the accident aggravated by the entry of the *coccus* gonorrhœa, but that the gonorrhœa infection formed a new and supervening source of injury which was the sole and independent cause of the loss of the eye.

